# Balancing Mass Fraction and Performance of Corn Husk/PLA Biocomposites for Moderate-Load Furniture Applications

**DOI:** 10.3390/polym18141772

**Published:** 2026-07-20

**Authors:** Fangmin Yuan, S. Siti Suhaily, Yuqing Wang

**Affiliations:** 1 School of Art and Design, Zhengzhou University of Industrial Technology, Zhengzhou 450000, China; 2 Product Design Department, School of The Arts, Universiti Sains Malaysia, Penang 11800, Malaysia; 3 Nusantara Institute of Creative Arts (INSAN), Universiti Teknologi MARA (UiTM), Shah Alam 40450, Malaysia

**Keywords:** corn husk, PLA, biocomposites, mass fraction, performance balance, moderate-load furniture applications

## Abstract

Corn husk is widely available as an agricultural by-product, yet its use in higher-value composite materials is still limited. This study examined how corn husk mass fraction affects the performance balance of corn husk/poly(lactic acid) (PLA) biocomposites (CHB) intended for moderate-load furniture board components. CHB containing 10 wt% and 30 wt% corn husk was prepared by hot pressing at 200 °C, 10 MPa, and 5 min. Density, water absorption, tensile, compressive, and flexural strength, Shore D hardness, and screw withdrawal resistance were evaluated. Increasing corn husk content from 10 wt% to 30 wt% reduced tensile strength, Shore D hardness, and screw withdrawal resistance. However, flexural strength increased from 22.8 ± 0.5 to 39.8 ± 1.1 MPa, while density decreased from 1.30 ± 0.03 to 1.09 ± 0.05 g/cm^3^. By contrast, 10 wt% CHB showed higher Shore D hardness and screw withdrawal resistance, reaching 84.1 ± 1.4 and 528.9 ± 22.9 N, respectively. The results indicate that 30 wt% CHB is more suitable for lightweight board components where flexural load-bearing is prioritized, whereas 10 wt% CHB is preferable for parts requiring higher surface hardness and screw connection reliability.

## 1. Introduction

The development of furniture materials from renewable resources has become increasingly relevant in green manufacturing and sustainable product design [1,2,3]. Beyond environmental considerations, however, materials intended for furniture use must also show measurable and reliable performance under service-related conditions [4]. Agricultural by-products provide a large and low-cost resource base for bio-based composite materials [5]. Corn husk, a common residue from corn production, is still mainly discarded, landfilled, burned, or used in low-value applications, and its conversion into higher-value composite products remains insufficient [6,7,8]. Using corn husk as a reinforcement in polymer composites may therefore provide a practical route for improving agricultural residue utilization while expanding the material options for sustainable furniture components.

Corn husk is a lignocellulosic material mainly composed of cellulose, hemicellulose, and lignin [9,10]. These components give it a certain structural rigidity and make it suitable for consideration as a natural reinforcement in particleboard, fiberboard, and bio-based composite systems [9,11]. Poly(lactic acid) (PLA), as a bio-based thermoplastic polymer, has good melt processability and molding stability, and can be combined with lignocellulosic fillers to form partially renewable composites [12]. In a corn husk/PLA system, however, the hydrophilic nature of corn husk and the relatively hydrophobic PLA matrix may restrict interfacial bonding, filler encapsulation, and stress transfer [13,14]. The formulation of corn husk/PLA biocomposites therefore needs to be adjusted carefully, rather than relying only on the addition of more biomass.

In natural-fiber-reinforced PLA composites, filler mass fraction is one of the main factors affecting material performance [13,15]. A lower fiber content may help preserve the continuity of the PLA matrix and improve the encapsulation of lignocellulosic particles. A higher fiber content, by contrast, can increase biomass utilization and reduce material density, and may also contribute to flexural load-bearing under certain conditions [16]. These potential benefits are often accompanied by trade-offs, including higher water uptake, more filler–matrix interfacial regions, and possible reductions in tensile, surface, or connection-related performance [17,18]. For furniture board components, this trade-off is particularly important because the material is expected to meet multiple requirements, including low density, limited water absorption, adequate mechanical strength, surface indentation resistance, and screw holding capacity.

Natural organic fillers, such as cork, cellulose fibers, wood flour, and other lignocellulosic residues, have been investigated as renewable reinforcements or functional fillers in polymer composites [12,19,20]. In contrast, current studies on corn husk-based polymer composites are still limited. Hasnan et al. [21] investigated the effect of corn husk content on the tensile properties of PLA/corn husk biocomposite films, while Chun et al. [22] studied corn husk fiber-reinforced epoxy composites in terms of mechanical properties and water absorption. These studies support the potential use of corn husk in polymer composites; however, they mainly focus on general material properties and do not fully address the combined performance requirements of furniture board components.

For such applications, material suitability is not determined by mechanical strength alone, but by the balance among density, moisture resistance, flexural load-bearing capacity, surface hardness, and fastening performance. In particular, screw withdrawal resistance has rarely been examined in corn husk/PLA systems, although it is directly relevant to furniture board components assembled with mechanical fasteners. Therefore, the novelty of this study lies in evaluating the mass-fraction-dependent performance balance of corn husk/PLA biocomposites for moderate-load furniture board components, with particular attention to Shore D hardness and screw withdrawal resistance in addition to conventional physical and mechanical properties. To address this gap, two representative CHB formulations containing 10 wt% and 30 wt% corn husk were prepared by hot pressing and evaluated in terms of density, water absorption, tensile, compressive and flexural strength, Shore D hardness, and screw withdrawal resistance.

## 2. Materials and Methods

### 2.1. Materials

The corn husk source and PLA matrix system were selected based on the authors’ previous work on the preparation of corn husk/PLA biocomposites (CHB) [23]. In the present study, the corn husk powder size was fixed at 120 mesh so that the influence of corn husk mass fraction on the physical, mechanical strength, and furniture-related properties of CHB could be examined.

Corn husks were collected from mature Zhengdan 985 corn plants grown in Zhengzhou, Henan Province, China. After collection, husk portions with obvious thickness variation, mechanical damage, or severe insect attack were removed. To ensure raw-material consistency, only the intact middle regions of the husks were retained for further processing. Corn husk is mainly composed of cellulose, hemicellulose, and lignin, together with small amounts of protein, ash, waxes, and other minor constituents [9,10].

Poly(lactic acid) (PLA, Ingeo^TM^ Biopolymer 4032D, NatureWorks LLC, Minnetonka, MN, USA) was selected as the thermoplastic matrix. This grade of PLA has suitable melt processability for hot-pressed natural-fiber-reinforced composites. Both PLA pellets and PLA powder were used. The pellets, with an average size of approximately 2.5 mm, served as the main matrix component. PLA powder was prepared by grinding pellets of the same grade to an average particle size of approximately 0.048 mm and was added to improve mixing and matrix distribution during hot pressing. According to the manufacturer’s technical data sheet, PLA 4032D has a melt flow rate of 14 g/10 min at 210 °C/2.16 kg, a glass transition temperature of 60–65 °C, and a melting temperature of 150–160 °C [24].

### 2.2. Pretreatment of Corn Husk Powder

After collection and selection, the corn husks were dried under naturally ventilated conditions, cut into small pieces, and then ground into powder using a high-speed grinder. The ground powder was sieved through a 120-mesh screen to obtain corn husk powder for composite preparation. Before hot pressing, the corn husk powder and PLA materials were pre-dried at 50 °C for 15 min to reduce the influence of residual moisture on the molding process and to lower the possibility of bubbles, pores, and interfacial defects [25].

### 2.3. Preparation of Corn Husk/PLA Biocomposites

Corn husk/PLA biocomposites (CHB) were prepared by hot pressing, with corn husk powder used as the reinforcing phase and PLA used as the thermoplastic matrix. To examine the effect of corn husk mass fraction, two CHB formulations containing 10 wt% and 30 wt% corn husk powder were prepared. These two mass fractions were selected as representative lower and higher filler-loading levels based on the previous literature and processing considerations, rather than as a full optimization series. The 10 wt% formulation represented a lower biomass content with better matrix continuity, whereas the 30 wt% formulation represented a higher biomass content with greater potential for density reduction and flexural load-bearing behavior. No lower corn husk contents below 10 wt% or intermediate loadings between 10 wt% and 30 wt% were investigated in this study. Therefore, the present results should be interpreted as a comparison between two selected formulations rather than as a complete filler-content optimization. A neat PLA reference sample was also prepared as the baseline for the unfilled matrix. All samples were hot-pressed at 200 °C, 10 MPa, and 5 min.

The detailed formulations are shown in Table 1. The PLA sample was prepared from 100 wt% PLA pellets and used as a neat matrix reference. In the two CHB formulations, the contents of PLA powder and CaCO_3_ were kept constant, while the corn husk powder content and PLA pellet content were varied. The CaCO_3_ used in this study was commercial ground calcium carbonate powder (GCC, micron-sized, untreated), which was added at 0.1 wt% as a minor processing additive to assist material flow and molding stability during hot pressing [26,27]. Because the main variable investigated in this study was the corn husk mass fraction rather than the CaCO_3_ content, CaCO_3_ was kept constant in the CHB formulations and was not treated as an independent reinforcing variable. Since CaCO_3_-specific control groups were not included, its independent contribution cannot be quantitatively separated from the present results.

PLA pellets served as the main matrix source and melted during hot pressing to form a continuous thermoplastic phase. PLA powder was used to improve material mixing and matrix distribution during hot pressing [28,29].

For the PLA reference sample, the pre-dried PLA pellets were weighed and directly placed in the mold. For the CHB samples, the pre-dried corn husk powder, PLA pellets, PLA powder, and CaCO_3_ were weighed according to Table 1 and mechanically mixed. The PLA pellets for the reference sample and the mixed CHB materials were then placed in the mold and hot-pressed at 200 °C and 10 MPa for 5 min. After cooling and demolding, the prepared plates were used for density, water absorption, mechanical strength, Shore D hardness, and screw withdrawal resistance tests.

### 2.4. Specimen Preparation

After hot pressing, the prepared plates were cooled, demolded, and edge-trimmed. Specimens were then cut to the required dimensions according to the different test items. To improve the comparability of the results, all specimens were taken from regions with stable molding quality and without visible bubbles, cracks, or edge defects. The cutting direction and specimen dimensions were kept consistent during specimen preparation. Before testing, the specimens were conditioned at room temperature to reduce the influence of machining and environmental differences on the test results. At least five replicate specimens were prepared for each test group, and the results were reported as mean ± standard deviation. Because the prepared plates had a thickness of 2 mm, some specimen dimensions were adjusted where necessary according to the available plate size. Therefore, the tests were conducted with reference to the listed standards, which were used as testing-method references rather than performance benchmarks, and the actual specimen dimensions, number of replicates, and reference methods are shown in Table 2.

### 2.5. Physical Property Tests

The physical properties of the prepared samples were mainly evaluated in terms of density and water absorption. Density was measured with reference to ISO 1183-1:2012 [30]. Before measurement, dust and loose edge debris were removed from the specimen surfaces, and the mass, length, width, and thickness of each specimen were recorded. The density was calculated from the ratio of mass to volume and was used to assess the effect of corn husk mass fraction on the lightweight characteristics of the composites. Density was calculated using Equation (1):

(1) $$\rho =\frac{m}{V}$$

where ρ  is the density (g/cm^3^), m  is the specimen mass (g), and V  is the specimen volume (cm^3^).

The water absorption test was used to evaluate the moisture uptake behavior of prepared samples under aqueous conditions and was conducted with reference to ISO 62:2008 [31]. Before testing, the specimens were dried in an oven to constant mass, and the initial dry mass was recorded. The specimens were then immersed in water at room temperature for 24 h. After immersion, the surface water was gently removed using absorbent paper, and the wet mass was measured immediately. Water absorption was calculated from the mass change before and after immersion, as shown in Equation (2):

(2) $$WA=\frac{m_{t}-m_{0}}{m_{0}}\times 100\%$$

where WA is the water absorption (%), m_0_ is the dry mass of the specimen before immersion (g), and m_t_ is the mass of the specimen after immersion (g).

### 2.6. Mechanical Strength Tests

The mechanical strength of the PLA reference samples and CHB was evaluated by tensile, compressive, and three-point flexural tests. All tests were carried out using a universal testing machine (AGS-X 10 kN, Shimadzu Corporation, Kyoto, Japan). Before testing, the specimens were kept at room temperature for 24 h to minimize the effects of specimen preparation and short-term environmental variation. During each test, the load–displacement response was recorded continuously, and the corresponding strength values were calculated according to the specimen dimensions. For each formulation and test item, at least five replicate specimens were tested, and the results are reported as mean ± standard deviation.

Tensile testing was performed with reference to ISO 527-2:2012 [32] at a crosshead speed of 1 mm/min. Tensile strength was taken as the maximum tensile stress reached before specimen failure.

Compressive testing was conducted with reference to ISO 604:2002 [33] at a crosshead speed of 1 mm/min. Because the hot-pressed plates prepared in this study were 2 mm thick, the compression specimen thickness followed the actual plate thickness. Compressive strength was defined as the maximum compressive stress reached before failure or the maximum stress recorded within the imposed strain limit of 60%, whichever occurred first.

Flexural strength was measured by three-point bending with reference to ISO 178:2019 [34]. The specimens were placed on two supports, and the load was applied at the mid-span position. The support span was set to 20 mm, and the crosshead speed was 1 mm/min. Flexural strength was defined as the maximum flexural stress reached during bending. The flexural test was included because bending resistance is closely related to the use of plate-like materials in moderate-load furniture board components.

### 2.7. Furniture-Related Performance Tests

To further evaluate the application suitability of the PLA reference samples and CHB for moderate-load furniture board components, Shore D hardness and screw withdrawal resistance tests were conducted. At least five replicate specimens were tested for each formulation and each test, and the results were reported as mean ± standard deviation.

Shore D hardness was measured with reference to ISO 868:2003 [35]. This test was used to evaluate the resistance of the sample surface to local indentation. Measurements were performed on flat and dry specimen surfaces. For each specimen, hardness was measured at several different positions, and the average value was taken as the hardness result of that specimen.

The screw withdrawal resistance test was conducted as a modified test with reference to EN 320:2011 [36]. This test was used to evaluate the local fastening performance of the samples when used as furniture board components. Before testing, a pilot hole with a diameter of 1.2 mm was drilled at the center of each specimen. A steel self-tapping screw with a diameter of 1.5 mm and a length of 10 mm was then inserted vertically into the specimen surface to an embedment depth of 2 mm, corresponding to the plate thickness. During testing, the specimen was fixed in the testing device, and a pull-out load was applied along the screw axis at a rate of 2 mm/min. The load change was continuously recorded until the screw was pulled out from the specimen or the connection failed. The maximum pull-out load reached during the test was used as the screw withdrawal resistance.

### 2.8. Statistical Analysis

All test results are expressed as mean ± standard deviation. Statistical analysis was performed for the mechanical strength data using one-way analysis of variance (ANOVA), followed by Tukey’s post hoc test for multiple comparisons among the PLA reference, 10 wt% CHB, and 30 wt% CHB groups. Differences were considered statistically significant at *p* < 0.05.

## 3. Results

### 3.1. Physical Appearance and Forming Quality of Corn Husk/PLA Biocomposites

Table 3 shows the visual appearance and forming quality of the CHB samples with different corn husk mass fractions. Both 10 wt% CHB and 30 wt% CHB were successfully formed into intact plate-like samples by hot pressing. No visible cracks, delamination, severe bubbles, or carbonization were observed on the sample surfaces, indicating that the selected processing conditions were suitable for preparing thin CHB panels.

The surface appearance changed with corn husk content. The 10 wt% CHB showed a relatively smooth and compact surface, which may be related to the higher PLA proportion and more continuous matrix coverage. By comparison, the 30 wt% CHB exhibited a more visible natural fiber texture and slightly higher surface heterogeneity. This change was expected because of the increased corn husk content, which can increase the proportion of biomass particles and filler–matrix interfacial regions in PLA-based composites [19,37]. However, the higher corn husk content did not prevent the formation of complete panels under the processing conditions used in this study.

### 3.2. Density and Water Absorption Behavior

Table 4 summarizes the density and 24 h water absorption of PLA and CHB with different corn husk mass fractions. The density of the PLA reference sample was 1.35 ± 0.05 g/cm^3^. After corn husk was incorporated, the density decreased to 1.30 ± 0.03 g/cm^3^ for 10 wt% CHB and further to 1.09 ± 0.05 g/cm^3^ for 30 wt% CHB. The reduction was more evident at 30 wt% corn husk content, showing that a higher biomass fraction contributed to lower material density. For furniture board components, this decrease is relevant because lower density can help reduce component weight, provided that the required mechanical and connection-related performance is maintained [38].

The water absorption of PLA was 0.27 ± 0.03%. The values increased to 0.37 ± 0.13% for 10 wt% CHB and 0.46 ± 0.05% for 30 wt% CHB. This increase was expected because corn husk contains hydrophilic components, especially cellulose and hemicellulose, whose hydroxyl groups can interact with water molecules [39]. A higher corn husk content may also create more filler–matrix interfacial regions and local moisture pathways within the composite [40].

Although 30 wt% CHB absorbed more water than PLA and 10 wt% CHB, its water absorption after 24 h immersion remained below 0.5%. This suggests that the PLA phase still limited rapid water penetration under the test conditions used here. From the physical-property perspective, 30 wt% CHB therefore offered lower density and higher corn husk utilization, with only a limited increase in short-term water absorption. Its application suitability, however, should be considered together with the mechanical strength and furniture-related properties discussed in the following sections.

### 3.3. Mechanical Strength of Corn Husk/PLA Biocomposites

Table 5 summarizes the tensile strength, compressive strength, and flexural strength of the PLA reference sample and corn husk/PLA biocomposites with different corn husk mass fractions.

Compared with the PLA reference sample, both CHB formulations showed lower tensile, compressive, and flexural strength. This reduction was mainly associated with the partial replacement of the continuous PLA phase by corn husk particles. The introduction of lignocellulosic particles increased the number of filler–matrix interfaces and may have reduced stress transfer efficiency, particularly under tensile and compressive loading.

The two CHB formulations showed different strength profiles. The 10 wt% CHB had higher tensile strength than the 30 wt% CHB, whereas the 30 wt% CHB showed much higher flexural strength. For compressive strength, the values of 10 wt% CHB and 30 wt% CHB were close, and no significant difference was observed between them. These results indicate that increasing the corn husk mass fraction did not improve or reduce all strength values in the same direction. Instead, the effect of mass fraction depended on the loading mode, which supports the need to evaluate CHB according to its intended furniture-related function.

#### 3.3.1. Tensile and Compressive Strength

For tensile strength, the PLA reference sample reached 50.0 ± 0.6 MPa, which was significantly higher than both CHB formulations. The tensile strength of 10 wt% CHB was 24.4 ± 0.8 MPa, significantly higher than that of 30 wt% CHB at 19.6 ± 0.6 MPa. The decrease in tensile strength after corn husk incorporation was mainly associated with the partial replacement of the continuous PLA matrix by corn husk particles. At 10 wt%, the PLA phase was still relatively sufficient to form a continuous matrix and encapsulate the corn husk particles, which was beneficial for tensile load transfer. When the corn husk content increased to 30 wt%, the relative PLA content decreased, while particle–particle contacts and filler–matrix interfacial regions increased. These changes may have promoted local stress concentration, interfacial defects, or less efficient load transfer, thereby reducing the tensile load-bearing capacity. Previous studies have also reported that the tensile performance of natural-fiber- or lignocellulosic-filler-reinforced PLA composites is commonly affected by filler dispersion, interfacial compatibility, and matrix continuity [41,42].

In this study, the lower tensile strength can be regarded as a performance compromise associated with the increased biomass fraction. Since no additional interfacial modification or local reinforcement was applied, the higher corn husk content may have limited tensile load transfer, although it also increased the renewable biomass content of the composite.

For compressive strength, the PLA reference sample reached 107.0 ± 1.4 MPa, which was significantly higher than both CHB formulations. The compressive strengths of 10 wt% CHB and 30 wt% CHB were 20.8 ± 1.6 MPa and 20.0 ± 0.9 MPa, respectively. Although 10 wt% CHB showed a slightly higher mean value, the difference between the two CHB formulations was not statistically significant. This suggests that, within the formulation range examined here, compressive strength was less sensitive to the increase in corn husk mass fraction than tensile strength. Previous studies have shown that the mechanical response of PLA or other polymer composites reinforced with lignocellulosic fillers is jointly affected by filler content, particle size, dispersion state, filler–matrix interfacial bonding, and loading mode [43,44]. For moderate-load furniture board components, 30 wt% CHB therefore showed lower tensile strength but maintained a basic compressive strength close to that of 10 wt% CHB.

#### 3.3.2. Flexural Strength

Flexural strength is important for plate-like furniture components because these parts are often subjected to bending during use, such as loads from self-weight, placed objects, and local connections [38]. As shown in Table 5, the PLA reference sample had the highest flexural strength, reaching 61.3 ± 0.7 MPa. Among the two CHB formulations, 30 wt% CHB showed significantly higher flexural strength than 10 wt% CHB, increasing from 22.8 ± 0.5 to 39.8 ± 1.1 MPa. This trend was different from the tensile results, where the higher corn husk content led to lower strength.

The higher flexural strength of 30 wt% CHB may be related to the different stress state during three-point bending. Under bending, the specimen is subjected to tensile, compressive, and shear stresses at the same time, so the response is not controlled by tensile load transfer alone. A higher corn husk content may allow for more particles to participate in resisting bending loads, as long as the PLA matrix can still provide sufficient support. Petchwattana and Covavisaruch [45] reported a similar improvement in the flexural performance of PLA/wood flour biocomposites within a certain filler-content range. Flexural behavior in natural-filler-reinforced polymer composites is also known to depend on filler content, dispersion, interfacial bonding, and matrix continuity [44].

Although 30 wt% CHB remained lower than PLA in flexural strength, its improvement over 10 wt% CHB is relevant to the intended application. Together with its lower density and higher biomass content, this result supports the use of 30 wt% CHB in selected moderate-load furniture board components where bending resistance and lightweight characteristics are prioritized.

Overall, the different mechanical responses of 10 wt% and 30 wt% CHB may be related to the balance between filler reinforcement and PLA matrix continuity. Previous studies have shown that filler content, filler dispersion, and fiber/matrix interfacial adhesion can strongly affect stress transfer and mechanical performance in natural-filler-reinforced PLA composites [12,41]. In this study, fracture surface morphology was not directly characterized; therefore, the detailed roles of void formation, crack initiation, and interfacial failure require further confirmation through future microscopic analysis.

### 3.4. Furniture-Related Performance

For furniture board components, surface hardness and screw holding capacity are closely related to service performance, especially in contact areas and mechanically fastened joints. Therefore, Shore D hardness and screw withdrawal resistance were measured to further evaluate the furniture-related performance of PLA and CHB. The results are summarized in Table 6. Among the tested materials, 10 wt% CHB showed higher Shore D hardness and screw withdrawal resistance, whereas 30 wt% CHB showed lower values for these two indicators. These results suggest that the two CHB formulations should be considered for different furniture-related functions rather than evaluated by a single performance index.

The difference between the bulk mechanical strength in Table 5 and the furniture-related properties in Table 6 can be mainly explained by the different mechanisms involved in these tests. Tensile, compressive, and flexural strengths depend on the load-bearing capacity of the whole specimen, including PLA matrix continuity, filler–matrix interfacial adhesion, and stress transfer [41]. The addition of corn husk particles may interrupt the continuous PLA phase and introduce local stress-concentration sites, leading to lower bulk strength than neat PLA. In contrast, Shore D hardness is more related to local indentation resistance [46], whereas screw withdrawal resistance is associated with friction and mechanical interlocking around the screw thread [47,48]. Therefore, for 10 wt% CHB, the relatively continuous PLA matrix and dispersed corn husk particles may have improved local hardness and screw-holding performance, even though the overall mechanical strength decreased. This improvement was not observed for 30 wt% CHB, suggesting that excessive corn husk addition may reduce matrix continuity and local support around the screw thread.

#### 3.4.1. Shore D Hardness

Shore D hardness was used to assess the resistance of the material surface to local indentation [46]. As shown in Table 6, 10 wt% CHB reached a Shore D hardness of 84.1 ± 1.4, slightly higher than that of the PLA reference sample at 81.0 ± 2.0. This improvement may be associated with the relatively continuous PLA matrix at the lower corn husk content, which allowed better particle encapsulation and surface consolidation. At the same time, a limited amount of corn husk particles may have contributed to local surface load-bearing. A similar increase in Shore D hardness was reported by Petchwattana and Covavisaruch [45] for PLA/wood flour biocomposites, suggesting that lignocellulosic fillers can improve surface hardness when their content remains within a suitable range.

By contrast, 30 wt% CHB showed a lower Shore D hardness of 77.4 ± 1.2. At this higher corn husk content, the reduced PLA fraction and the increased number of filler–matrix interfaces may have lowered the local uniformity of the surface structure. This could make the material more susceptible to indentation under the Shore D test. Narlıoğlu et al. [49] also reported that the hardness of wood sawdust/PLA composites did not increase continuously with filler content, as higher filler levels may reduce dispersion quality or promote particle agglomeration. In the present study, increasing the corn husk content from 10 wt% to 30 wt% therefore did not further improve surface hardness; instead, 10 wt% CHB showed better resistance to local indentation.

#### 3.4.2. Screw Withdrawal Resistance

Screw withdrawal resistance was measured to evaluate the ability of the material to hold mechanical fasteners, which is directly related to the connection reliability of furniture board components. Yorur et al. [47] reported that screw withdrawal resistance is affected by several factors, including panel type, screw direction, pilot holes, and adhesive use. As shown in Table 6, 10 wt% CHB reached 528.9 ± 22.9 N, higher than the PLA reference sample at 467.7 ± 38.3 N. This improvement may be attributed to the relatively continuous PLA matrix and the dispersed corn husk particles around the screw. Under this formulation, the PLA phase could still provide local support, while the corn husk particles may have increased friction and mechanical interlocking at the screw–material interface.

By contrast, 30 wt% CHB showed a lower screw withdrawal resistance of 406.9 ± 23.7 N. The higher corn husk content reduced the relative amount of PLA matrix and increased the number of filler–matrix interfacial regions, which may have weakened the local support around the screw. A less uniform structure around the screw hole could also reduce the effectiveness of mechanical interlocking during withdrawal. Borysiuk et al. [48] similarly reported that the matrix/filler ratio is an important factor affecting screw holding performance in PLA-based wood–plastic composite panels, and that higher lignocellulosic filler content may reduce screw holding capacity.

These results show that 10 wt% CHB is more suitable for furniture parts where screw connection reliability is required, such as fixing points, joint areas, or components subjected to repeated fastening. For 30 wt% CHB, the lower screw withdrawal resistance suggests that surface treatment, connection design optimization, or local reinforcement would be needed if the material is used in areas with high screw-holding requirements.

### 3.5. Mass Fraction–Performance Balance and Application Suitability

The results above show that changing the corn husk mass fraction shifted the performance profile of CHB rather than improving all properties in the same direction. For furniture board components, material suitability should be judged by a combination of density, water absorption, mechanical strength, surface hardness, screw withdrawal resistance, and biomass content. Based on this combined evaluation, 10 wt% CHB and 30 wt% CHB correspond to different furniture-related requirements, as summarized in Table 7.

The 10 wt% CHB showed clearer advantages in Shore D hardness and screw withdrawal resistance. This formulation is therefore more suitable for local furniture parts where surface indentation resistance and screw connection reliability are required, such as fixing points, joint areas, or small contact components. The result is consistent with the relatively higher PLA proportion, which may help maintain matrix continuity and local support around the surface or screw hole.

The 30 wt% CHB, by contrast, showed lower surface hardness and screw withdrawal resistance, but it had higher flexural strength, lower density, and higher corn husk content. This makes it more appropriate for selected moderate-load board components where bending resistance and weight reduction are more important than screw holding performance. Typical examples may include small tabletops, side panels, shelves, or non-high-load panels, provided that the connection areas are properly designed.

Therefore, CHB should be considered as a function-specific candidate material for moderate-load furniture board components rather than as a complete replacement for conventional wood-based panels. For parts requiring higher screw holding capacity or surface indentation resistance, further improvement through surface treatment, connection design optimization, or local reinforcement would still be necessary.

## 4. Conclusions

This study evaluated 10 wt% and 30 wt% corn husk/PLA biocomposites (CHB) prepared by hot pressing at 200 °C, 10 MPa, and 5 min. Both formulations formed intact plate-like samples without visible cracking, delamination, severe bubbling, or carbonization, indicating that the selected processing conditions were suitable for preparing thin CHB panels.

The results show that changing the corn husk mass fraction adjusted the performance balance of the composites, rather than producing a single formulation that was superior in all aspects. Increasing the corn husk content from 10 wt% to 30 wt% reduced the density from 1.30 ± 0.03 to 1.09 ± 0.05 g/cm^3^ and improved the flexural strength from 22.8 ± 0.5 to 39.8 ± 1.1 MPa, although water absorption increased slightly. By contrast, the 10 wt% CHB showed better tensile performance, Shore D hardness, and screw withdrawal resistance. These findings suggest that the filler content should be selected according to the intended furniture function, rather than simply maximizing the biomass fraction.

From an application perspective, the 10 wt% CHB is more suitable for connection zones, fixing points, and surface-contact parts where indentation resistance and screw holding are important. The 30 wt% CHB is more appropriate for selected moderate-load board components where flexural load-bearing capacity, lower density, and higher bio-based content are prioritized. The main contribution of this study is therefore to demonstrate a function-oriented approach for balancing biomass content and performance in corn husk/PLA composites for moderate-load furniture applications. However, this study was limited to two corn husk mass fractions and mainly focused on macroscopic physical, mechanical, and furniture-related properties. Further work should investigate interfacial bonding, filler dispersion, void formation, crack initiation, fracture surface morphology, surface treatment, long-term durability, dimensional stability, and component-level validation to confirm the broader applicability of CHB in furniture products.

## Figures and Tables

**Table 1:** Formulations of the PLA reference and corn husk/PLA biocomposites.

Sample Code	Corn Husk Powder (wt%)	PLA Pellets (wt%)	PLA Powder (wt%)	CaCO_3_ (wt%)	Role in Comparison
PLA	0	100.0	0	0	Neat PLA reference without corn husk
10 wt% CHB	10.0	80.0	9.9	0.1	Lower corn husk content; matrix continuity and connection-related performance
30 wt% CHB	30.0	60.0	9.9	0.1	Higher corn husk content; density reduction, biomass utilization, and flexural behavior

**Table 2:** Specimen dimensions, number of replicates, and reference methods for each test item.

Test item	Specimen Dimensions (mm)	Number of Specimens	Reference Method
Density	70 × 10 × 2	*n* = 5	With reference to ISO 1183-1 [30]
Water absorption	70 × 10 × 2	*n* = 5	With reference to ISO 62 [31]
Tensile test	70 × 10 × 2	*n* = 5	With reference to ISO 527-2 [32]
Compression test	10 × 10 × 2	*n* = 5	With reference to ISO 604 [33]
Flexural test	70 × 10 × 2	*n* = 5	With reference to ISO 178 [34]
Shore D hardness	70 × 10 × 2	*n* = 5	With reference to ISO 868 [35]
Screw withdrawal resistance	80 × 80 × 2	*n* = 5	With reference to EN 320:2011 [36]

Specimen dimensions are expressed as length × width × thickness.

**Table 3:** Visual appearance and forming quality of corn husk/PLA biocomposites with different mass fractions.

Sample	Visual Appearance	Forming Quality	Possible Explanation	Process Feasibility
10 wt% CHB	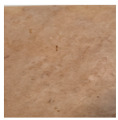	Relatively smooth and compact surface; complete plate structure; no obvious cracks, delamination, severe bubbles, or carbonization observed.	The higher PLA content may have helped form a more continuous matrix and improve the encapsulation of corn husk particles.	Good formability
30 wt% CHB	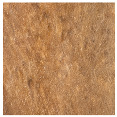	More visible natural fiber texture and biomass-related surface features; complete plate structure; no obvious cracks, delamination, severe bubbles, or carbonization observed.	The higher corn husk content increased the visible biomass texture and surface heterogeneity, while the PLA matrix still provided basic coverage and bonding.	Formable, with a rougher surface

**Table 4:** Density and water absorption of PLA and corn husk/PLA biocomposites with different mass fractions.

Sample	Density (g/cm^3^)	Water Absorption (%)
PLA	1.35 ± 0.05	0.27 ± 0.03
10 wt% CHB	1.30 ± 0.03	0.37 ± 0.13
30 wt% CHB	1.09 ± 0.05	0.46 ± 0.05

Values are expressed as mean ± standard deviation, *n* = 5.

**Table 5:** Mechanical strength of PLA and corn husk/PLA biocomposites with different mass fractions.

Property	PLA	10 wt% CHB	30 wt% CHB
Tensile strength (MPa)	50.0 ± 0.6 ᵃ	24.4 ± 0.8 ᵇ	19.6 ± 0.6 ᶜ
Compressive strength (MPa)	107.0 ± 1.4 ᵃ	20.8 ± 1.6 ᵇ	20.0 ± 0.9 ᵇ
Flexural strength (MPa)	61.3 ± 0.7 ᵃ	22.8 ± 0.5 ᶜ	39.8 ± 1.1 ᵇ

Values are expressed as mean ± standard deviation, *n* = 5. Different superscript letters within the same row indicate significant differences among groups (*p* < 0.05).

**Table 6:** Furniture-related properties of PLA and corn husk/PLA biocomposites.

Material	Shore D Hardness	Screw Withdrawal Resistance (N)
PLA	81.0 ± 2.0	467.7 ± 38.3
10 wt% CHB	84.1 ± 1.4	528.9 ± 22.9
30 wt% CHB	77.4 ± 1.2	406.9 ± 23.7

Values are expressed as mean ± standard deviation, *n* = 5.

**Table 7:** Performance balance and suggested application directions of 10 wt% and 30 wt% CHB.

Sample	Main Advantages	Main Limitations	Suggested Application Direction
10 wt% CHB	Higher Shore D hardness; higher screw withdrawal resistance; lower water absorption than 30 wt% CHB	Lower flexural strength and lower corn husk content than 30 wt% CHB	Connection zones, fixing points, contact areas, or small components where surface hardness and screw holding are important
30 wt% CHB	Higher flexural strength; lower density; higher corn husk utilization	Lower Shore D hardness and screw withdrawal resistance than 10 wt% CHB	Selected moderate-load furniture board components where bending resistance, lower weight, and higher biomass content are prioritized

## Data Availability

The data supporting the findings of this study are available from the corresponding author upon reasonable request.

## Abbreviations

The following abbreviations are used in this manuscript:

CH	Corn husk
CHB	Corn husk/PLA biocomposites
PLA	Polylactic acid
